# Acute Kidney Injury Induced by *Bothrops* Venom: Insights into the Pathogenic Mechanisms

**DOI:** 10.3390/toxins11030148

**Published:** 2019-03-05

**Authors:** Polianna Lemos Moura Moreira Albuquerque, Geraldo Bezerra da Silva Junior, Gdayllon Cavalcante Meneses, Alice Maria Costa Martins, Danya Bandeira Lima, Jacques Raubenheimer, Shihana Fathima, Nicholas Buckley, Elizabeth De Francesco Daher

**Affiliations:** 1 Medical Sciences of Post-Graduate Program, Department of Internal Medicine, Federal University of Ceara, CEP 60416-200 Fortaleza, Ceara, Brazil; gdayllon@yahoo.com.br (G.C.M.); ef.daher@uol.com.br (E.D.F.D.); 2 School of Medicine, Public Health and Medical Sciences Post-Graduate Programs, School of Medicine, University of Fortaleza, CEP 60811-905 Fortaleza, Ceara, Brazil; geraldobezerrajr@yahoo.com.br; 3 Center for Toxicological Assistance, Dr. José Frota Institute, CEP 60025-061 Fortaleza, Ceara, Brazil; 4 Pharmacology and Pharmaceutial Sciences Post-Graduate Programs, Federal University of Ceara, CEP 60430-275 Fortaleza, Ceara, Brazil; martinsalice@gmail.com (A.M.C.M.); danya.dbl@gmail.com (D.B.L.); 5 Clinical Pharmacology, Sydney Medical School, University of Sydney, Sydney, NSW 2006, Australia; jacques.raubenheimer@sydney.edu.au (J.R.); fathimashihana20@gmail.com (S.F.); nicholas.buckley@sydney.edu.au (N.B.)

**Keywords:** *Bothrops*, envenomation, acute kidney injury, renal tubular dysfunction, coagulopathy, novel biomarkers

## Abstract

Acute kidney injury (AKI) following snakebite is common in developing countries and *Bothrops* genus is the main group of snakes in Latin America. To evaluate the pathogenic mechanisms associated with *Bothrops* venom nephrotoxicity, we assessed urinary and blood samples of patients after hospital admission resulting from *Bothrops* snakebite in a prospective cohort study in Northeast Brazil. Urinary and blood samples were evaluated during hospital stay in 63 consenting patients, divided into AKI and No-AKI groups according to the KDIGO criteria. The AKI group showed higher levels of urinary MCP-1 (Urinary monocyte chemotactic protein-1) (median 547.5 vs. 274.1 pg/mgCr; *p* = 0.02) and urinary NGAL (Neutrophil gelatinase-associated lipocalin) (median 21.28 vs. 12.73 ng/mgCr; *p* = 0.03). Risk factors for AKI included lower serum sodium and hemoglobin levels, proteinuria and aPTT (Activated Partial Thromboplastin Time) on admission and disclosed lower serum sodium (*p* = 0.01, OR = 0.73, 95% CI: 0.57–0.94) and aPTT (*p* = 0.031, OR = 26.27, 95% CI: 1.34–512.11) levels as independent factors associated with AKI. Proteinuria showed a positive correlation with uMCP-1 (r = 0.70, *p* < 0.0001) and uNGAL (r = 0.47, *p* = 0.001). FE_Na_ (Fractional Excretion of sodium) correlated with uMCP-1 (r = 0.47, *P* = 0.001) and uNGAL (r = 0.56, *p* < 0.0001). sCr (serum Creatinine) showed a better performance to predict AKI (AUC = 0.85) in comparison with new biomarkers. FE_K_ showed fair accuracy in predicting AKI (AUC = 0.92). Coagulation abnormality was strongly associated with *Bothrops* venom-related AKI. Urinary NGAL and MCP-1 were good biomarkers in predicting AKI; however, sCr remained the best biomarker. FE_K_ (Fractional Excretion of potassium) emerged as another diagnostic tool to predict early AKI. Positive correlations between uNGAL and uMCP-1 with proteinuria and FE_Na_ may signal glomerular and tubular injury. Defects in urinary concentrations highlighted asymptomatic abnormalities, which deserve further study.

## 1. Introduction

Snakebite-related acute kidney injury (AKI) is a common complication following envenomation by snakes of the genus *Bothrops*, of which incidence varies between 1.4 and 38.5% [1,2,3,4,5]. Most snakebites in Latin America are caused by *Bothrops* snakes [6]. In Brazil, there are the following *Bothrops* species that are medically important: *B. jararaca* (South and Southeast), *B. moojeni* (Center-West), *B. atrox* (North) and *B. erythromelas* (Northeast) [4,7]. Snake venom metalloproteinases (SVMPs) and serine proteinases (SVSPs) are the main toxins with hemotoxic properties [8,9]. Renal tubular injury may be due to mechanical obstruction by red blood cell casts and cytotoxic effects of oxidative stress induced by hemoglobin, heme or iron released from red blood cells [10].

The most common coagulation syndromes related to snake envenomation are venom-induced consumption coagulopathy (VICC), thrombotic microangiopathy (TMA) and disseminated intravascular coagulation (DIC) [11,12]. Some studies have reported factor deficiencies in *Bothrops* envenomation, such as fibrinogen, fibrinogen degradation products, D-dimer and α-2-antiplasmin [13]. Results in rats with coagulopathy induced by moojenactivase (MooA), a procoagulant *Bothrops* venom metalloprotease, correlated DIC with renal microthrombin deposits and end-organ failure [12,14].

AKI in *Bothrops* envenomation is oliguric, severe and early [15]. Few studies have reported novel renal biomarkers in *Viperid* snakebites and none in *Bothrops* snakes [16,17,18,19]. The present study investigates *Bothrops* venom-related AKI and the pathophysiological mechanisms based on coagulation disturbances, novel biomarkers and renal tubular dysfunction in Northeast Brazil.

## 2. Results

**Figure 1:**
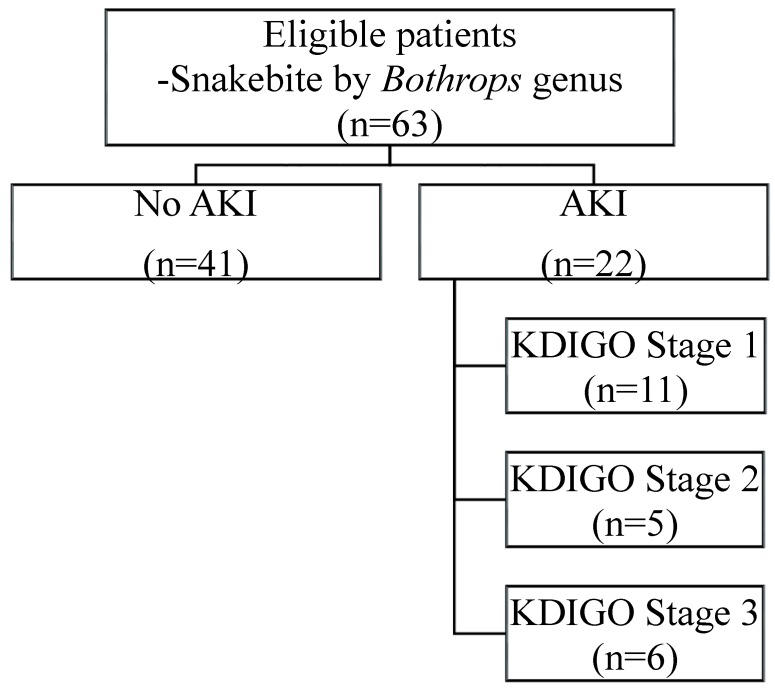
Patient recruitment profile. Data expressed as number of patients (n).

Of the 63 patients included in the study, 22 (34.9%) developed AKI, with varying degrees of severity according to the KDIGO criteria [20] (Figure 1).

Demographic and clinical variables were similar in the AKI and No-AKI groups, except for hospital length of stay, which was longer in the AKI group (*p* = 0.003) (Table 1). Hemorrhagic symptoms and hypotension were not reported. One patient required hemodialysis and five patients showed partial recovery of renal function at discharge. Antihistamines and corticoids were used before the antivenom administration.

The AKI group showed lower levels of averaged hemoglobin (median 12.28 vs. 13.4 g/dL, *p* = 0.03) and averaged hematocrit (median 35.1 vs. 38.55%, *p* = 0.019). The nadirs of hemoglobin and hematocrit (lowest values) during hospital stay were lower in the AKI group (Table 2). Leukocytes, platelet count, serum creatine kinase, glucose, albumin and potassium levels were similar in the AKI and No-AKI groups (Table 2). The nadir of serum sodium was lower in the AKI group (median 139 vs. 142 mEq/L; *p* = 0.02) (Table 2).

Baseline sCr was similar in the AKI and No-AKI groups (median 0.85 vs. 0.8 mg/dL; *p* = 0.23). The AKI group showed higher levels of averaged serum urea (median 45.1 vs. 34 mg/dL; *p* = 0.004) and sCr on admission (median 1.45 vs. 0.9 mg/dL; *p* ≤ 0.0001). The estimation of the glomerular filtration rate (eGFR) on admission was lower in the AKI group (mean 55.45, SD 6.99 vs. 99.31, SD 3.91 mL/min per 1.73 m^2^; *p* < 0.0001). Proteinuria normalized by urinary creatinine was higher in the AKI group (median 643 vs. 260 mg/gCr; *p* = 0.03) (Table 2).

Fractional excretion of potassium was higher in the AKI group (14.54 vs. 8.64%; *p* < 0.0001). FE_Na_, FE_Cl_, FE_U_, Uosm (Urine Osmolality), Posm (Plasmatic Osmolality), TTKG (Transtubular Potassium Concentration Gradient) and the Uosm/Posm ratio were similar in the AKI and No-AKI groups. However, Posm (normal range, 275–295 mOsm/kg H_2_O) and Uosm/Posm (ratio < 2.8) showed abnormal values in both groups (Table 2).

Activated Partial Thromboplastin Time (aPTT) in the AKI group on admission revealed a higher number of patients with abnormal (6/22, 27% vs. 4/41, 9.8%) and incoagulable blood tests (14/22, 63.6% vs. 20/41, 48.7%) (*p* = 0.03). aPTT in the AKI group, including abnormal and incoagulable blood tests together, remained higher than in the No-AKI group (20/22, 90.6% vs. 24/41, 58.5%; *p* = 0.01). The same comparisons with PT were not significant.

**Figure 2:**
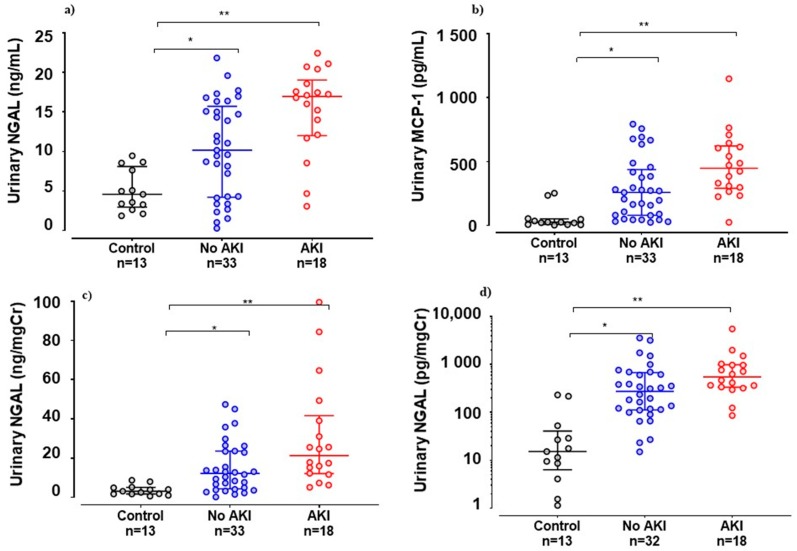
Biomarker levels after snakebite. (**a**) Urinary NGAL non-normalized by urinary creatinine; (**b**) urinary MCP-1 levels non-normalized by urinary creatinine; (**c**) urinary NGAL normalized by urinary creatinine; (**d**) urinary 107 MCP-1 levels normalized by urinary creatinine. * *p* < 0.05, according to the Mann–Whitney-U test. ** *p* < 0.0001, according to 1-way ANOVA and the Kruskal–Wallis test to compare between the three groups and the Mann–Whitney-U test between controls and the No-AKI group. Control: n = 13; No-AKI: n = 41; AKI group: n = 22.

The AKI group showed higher levels of urinary MCP-1 (median 547.5 vs. 274.1 pg/mgCr; *p* = 0.02) and urinary NGAL (median 21.28 vs. 12.73 ng/mgCr; *p* = 0.03). Serum NGAL on admission was similar in both groups (Figure 2).

Multivariate analysis with logistic regression included lower serum sodium (mEq/L), lower hemoglobin (g/dL), proteinuria (mg/gCr) and aPTT on admission (normal vs. abnormal/incoagulable) and disclosed lower serum sodium (*p* = 0.01, OR = 0.73, 95% CI: 0.57–0.94) and aPTT levels (*p* = 0.031, OR = 26.27, 95% CI: 1.34–512.11) as independent factors associated with AKI (Table 3).

**Figure 3:**
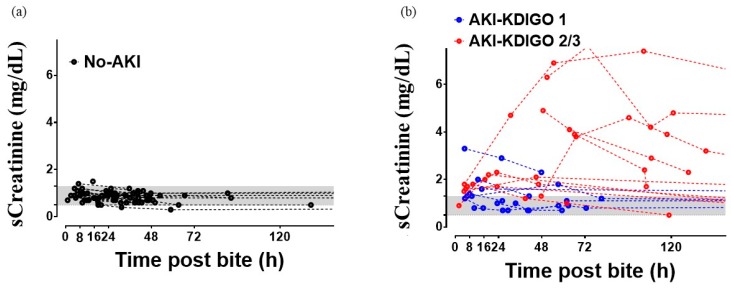
Kinetic serum creatinine levels (sCreatinine) after snakebite in the No-AKI group (**a**) and AKI group (**b**). The gray-shaded area illustrates the normal range of serum creatinine.

Serial sCr in the No-AKI group did not show any variability during the post-bite period (Figure 3a). The AKI group showed considerable changes during the post-bite period regarding KDIGO stages 1, 2/3 (Figure 3b).

Proteinuria normalized by urinary creatinine showed a positive correlation with uMCP-1 (rho = 0.70, *p* < 0.0001) and uNGAL (rho = 0.47, *p* = 0.001) (Table 4). FE_Na_ correlated with uMCP-1 (rho = 0.47, *p* = 0.001) and uNGAL (rho = 0.56, *p* < 0.0001). FE_K_ did not correlate with these biomarkers (Table 3).

**Figure 4:**
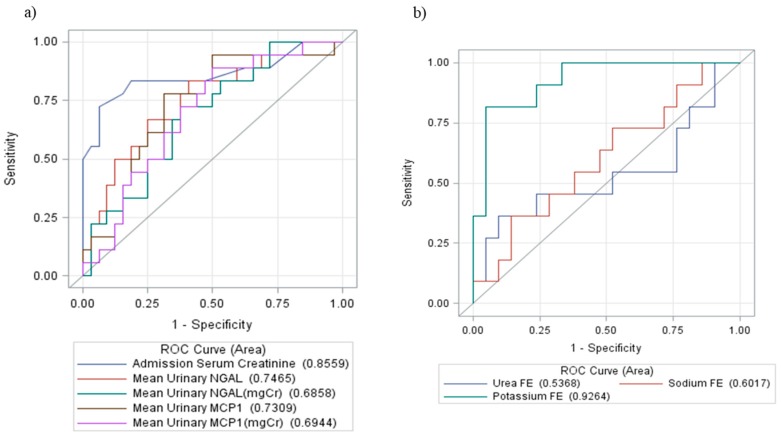
Receiver operating characteristic (ROC) curves for: (**a**) serum creatinine and urinary biomarkers and (**b**) fractional excretion of urea, potassium and sodium, on hospital admission after *Bothrops* envenomation to predict AKI. Urea FE, Fractional Excretion of urea. Sodium FE, Fractional Excretion of sodium. Potassium FE, Fractional Excretion of potassium.

The comparison of uMCP-1 and uNGAL levels in the patient group with the healthy controls revealed a gradual increase (*p* < 0.0001) (Figure 2). Receiver operating curves were constructed to display true positive and false positive rates of AKI on admission. sCr showed better performance (area under the curve or AUC = 0.85) in comparison with new biomarkers (Figure 4). FE_K_ showed fair accuracy in predicting AKI (AUC = 0.92) (Figure 4).

**Figure 5:**
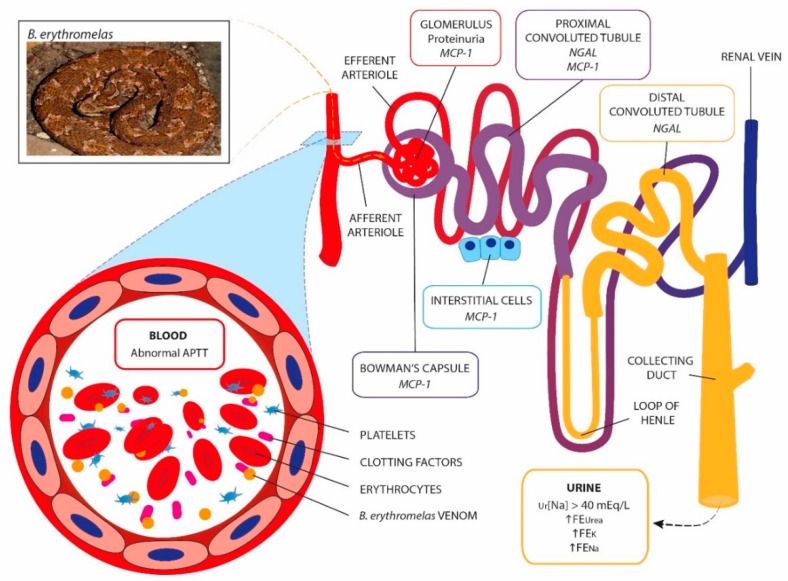
Proposed pathogenic AKI mechanisms following *Bothrops* envenomation. A consistent association between abnormal/incoagulable aPTT with AKI development presumes renal microvasculature impairment as an important step in the pathophysiology of AKI. High energy demand with relatively low net oxygen extraction make the kidneys susceptible to vascular perfusion and oxygenation impairment. The damaged microcirculation leads to hypoxia and oxidative stress. Thus, the injured microvascular endothelium and glycocalyx alterations lead to endothelial cell activation with new expression of cell surface markers, which might promote an increase of MCP-1 secreted by mononuclear leukocytes, cortical tubular epithelial cells and podocytes. Increases of urinary NGAL and MCP-1 are associated with renal inflammation, glomerular damage and tubular atrophy, mainly in proximal and distal convoluted tubules. Interstitial nephritis associated with an increase of MCP-1 could not be removed and may likely worsen blood flow in the microcirculation, contributing to AKI. In this study, proteinuria signaled glomerular damage related to *Bothrops* venom. Abnormalities in FEK, FENa, FEUrea and defects in urinary concentrations suggested tubular atrophy. Moreover, Ur [Na] > 40 mEq/L is another sign of acute tubular necrosis.

The pathogenic mechanisms of AKI following *Bothrops* envenomation and the role of the novel biomarkers and coagulation abnormalities were proposed (Figure 5).

## 3. Discussion

*B. erythromelas* is the main species responsible for snakebite envenomation in Northeast Brazil (approximately 90% of *Bothrops* envenomation events), although some cases have been ascribed to *B. neuwiedi* but were never confirmed [6,21]. Moreover, *B. erythromelas* shows similar taxonomic characteristics to the *B. neuwiedi* group [22]. The overall toxin profile of *Bothrops erythromelas* venom explains the main reported local and systemic effects. In tropical countries, snakebites affect young and economically active adults, without pre-existing comorbidities and farmers from rural communities [21,22]. A long period elapsed between the snakebite and medical care/antivenom administration represented an example of a weak health system in inhospitable areas [23].

Lower hemoglobin levels in the AKI group suggest a contribution of the hemotoxic mechanism following *Bothrops* venom-related AKI. The proteomic analysis of *B. erythromelas* venom revealed a predominance of SVMPs, P-III class [21]. Berythractivase, P-III SVMP, the only one characterized from *B. erythromelas*, is non-hemorrhagic and capable of triggering endothelial procoagulant and proinflammatory cell responses, contributing to the depletion of circulating clottable fibrinogen, synergistically potentiating the hemorrhagic activity of P-III SVMPs and thus increasing the incidence of systemic bleeding [21]. *B. erythromelas* venom in Ceara state, where our study was performed, shows <0.1% of phospholipase B (PLB) in its overall protein composition, which contributes with direct hemolytic activity found in the *Bothrops* genus venom [21]. Hemorrhagic abnormalities in *Bothrops* envenomation cases have been independently associated with AKI development in a previous study [24]. Most patients in the present study did not develop AKI (70%) or had AKI KDIGO stages 1/2 (25%), with mild severity; thus, there was no evidence of hemorrhagic symptoms.

Thrombocytopenia was not associated with AKI development. Although an abnormal aPTT has been associated with AKI development, the study did not show any differences in platelet levels between the AKI and No-AKI groups. A large number of constituents from snake venoms can inhibit or activate platelet aggregation, such as SVMPs, SVSPs, phospholipase A2 (PLA2), disintegrins, L-amino acid oxidases (LAAOs), C-type lectins-like and 5’-nucleotidases [25]. A group of PLA2 from *B. pauloensis* venom was isolated from *B. erythromelas* venom and it can inhibit platelet aggregation [26]. An insignificant amount of SVSPs, referred to as TLE, in *B. erythromelas* from Ceara, in comparison with the main species of *Bothrops*, might cause the absence of thrombocytopenia, as TLEs induce platelet release and aggregation [8,21,26]. The interactions between venom compounds with coagulant factors impaired the physiological role of platelets. Snake venom C-type lectins reduce platelet function with or without thrombocytopenia, inhibiting surface receptors, such as the von Willebrand receptor, collagen receptor and integrin α2β1 [21].

The higher incidence of abnormal/incoagulable aPTT in the AKI group points to the contribution of the intrinsic coagulation impairment pathway in the *Bothrops* venom-related AKI mechanism. Despite the abnormalities in coagulation tests, many patients with VICC exhibit minimal clinical features [11]. This study did not report severe bleedings or fatalities due to *Bothrops* snakebite. In the comparative analysis of coagulating activities of *Bothrops* venom, *B. erythromelas* venom showed the highest levels of factor X and prothrombin activators without thrombin-like activity. A recent study highlighted the lower efficiency of the *Bothrops* antivenom manufactured in Brazil in neutralizing Factor X-activating toxins compared to prothrombin-activating toxins, which might have an impact on the clinical picture [27]. Multivariate analysis showed that abnormal/incoagulable aPTT is a risk factor for AKI development.

The hypervolemic status in oliguric *Bothrops* venom-related AKI contributes to the lower levels of sodium and hematocrit in the AKI group, due to a dilutional effect. Hyponatremia has been reported in Russell’s Viper syndrome and is considered an independent factor associated with AKI [28]. On admission, our patients commonly showed hypervolemic signals and symptoms after a long period post-bite in both groups. Bradykinin-potentiating-peptide 13a (BPP13a), an inhibitor of RAAS, which enhances the hypotensive effect of circulating bradykinin, was isolated from *B. erythromelas* venom and accounted for just 1.1 ± 0.3% of the total venom proteins in a recent proteomic study [21,29]. In the current study, hypotension likely occurred before hospital admission and it was not reported here because of the long period post-bite. Normal levels of serum potassium and TTKG in both groups (<6) suggested no RAS activation [30]. TTKG is used to gauge renal potassium secretion by the cortical-collecting duct, indirectly assessing mineralocorticoid bioactivity.

Creatine kinase levels did not show significant differences between the AKI and No-AKI groups. Rhabdomyolysis was not a decisive mechanism of kidney injury in *Bothrops* envenomation. Myonecrosis may be explained by phospholipid hydrolysis causing disruption of the plasma membrane of skeletal muscle myocytes and a large influx of Ca^2+^ in muscle cells [21]. However, few studies have reported high levels of creatine kinase in *Bothrops* snakebites [31].

Hematuria and proteinuria are common clinical renal manifestations in snakebites and provide insights into kidney damage [9]. This is the first clinical study evaluating proteinuria in *Bothrops* envenomation. The AKI group showed higher levels of proteinuria, indicating glomerular changes. Glomerular damage was demonstrated for the first time in an animal model after intravenous administration of *B. moojeni* venom in rats [32]. *B. moojeni* venom caused proteinuria and ultrastructural changes in the visceral epithelium and glomerular capillary tufts compatible with the renal dysfunction described, contributing to nephrotoxicity [32]. In another study, Habu snake venom (HSV)-induced glomerulonephritis showed a progressive course of mesangial cell migration, proliferation, mononuclear cell infiltration and extracellular matrix accumulation in cultured mesangial cells in vitro. MCP-1 messenger RNA (mRNA) levels in cultured mesangial cells were increased through stimulation with HSV, even during the recovery time [17]. In our study, proteinuria strongly correlated with high levels of MCP-1.

Higher levels of urinary MCP-1 in the AKI group reflect another pathogenic pathway in *Bothrops* envenomation. MCP-1 is a low molecular weight CC-chemokine (13KDa) associated with innate immunity, easily filtered into the urine. The first recruitment step seems to be systemic inflammatory conditions. Secondly, MCP-1 is released at sites of inflammation and stored in the local glycocalyx. Thirdly, local MCP-1 production leads to the release of inflammatory cytokines, differentiation and recruitment of monocytes/macrophages [33].

Urinary MCP-1 positively correlated with FE_Na_. MCP-1 is secreted by mononuclear leukocytes, cortical tubular epithelial cells and podocytes and is implicated in renal inflammation, glomerular damage, tubular atrophy and fibrosis [34,35]. However, the location of MCP-1 expression was found to be predominantly in tubular cells and not in glomeruli [33]. The increase of FE_Na_ in accordance with urinary MCP-1 represents tubular impairment due to AKI in *Bothrops* envenomation.

Urinary NGAL was higher in the AKI group, which reflects structural kidney injury. NGAL is a small siderophore protein intensely up-regulated and excreted in acute tubular damage [36]. It can be detected in plasma and urine in the early phases of AKI, being readily filtered in the glomerulus and reabsorbed in the proximal tubular segments [36]. It might be a more specific AKI marker in patients with systemic inflammation, when multi-organ damage is less pronounced [37]. In the current study, serum NGAL did not show a significant difference between the groups, likely because all subjects showed the same systemic effects. However, urinary NGAL showed increased levels in the AKI group in comparison with healthy controls and the No-AKI group. Moreover, urinary NGAL positively correlated with FE_Na_ and proteinuria, which emphasizes the association with acute tubular injury.

The fractional excretion of potassium was higher in the AKI group and accurate in predicting AKI. However, there were no differences between FE_Na_ and FE_Ur_. Although FE_Ur_ and FE_Na_ may be useful in differentiating between functional (pre-renal) and structural AKI (secondary to ischemia, toxins or both), the interpretation of these parameters remains conflicting [38,39,40,41]. Few studies have reported the performance of FE_K_ in predicting AKI [40,41]. FE_K_ was analyzed in patients with different diagnoses in an intensive care unit, with No-AKI, transient and persistent AKI without renal replacement therapy. It was not altered by diuretic use [41]. In our study, FE_K_ showed the highest accuracy by the AUC-ROC in predicting AKI diagnosis. Most urinary K can be accounted by electrogenic secretion, mediated by principal cells in the initial and cortical collecting duct, leading to greater and more evident variations in FE_K_ than FE_Na_ or FE_urea_ [42]. K secretion is partially dependent on the luminal tubular flow rate, which was not measured between the groups; however, urinary volume is not included in FE_K,_ which goes against this idea [41]. FE_Na_ was higher than 1% and FE_urea_ was higher than 35% in the AKI group, which could represent impairment of the tubular capacity to retain sodium and urea. This fact could interfere with renal K handling, which is enhanced by Na reabsorption, stimulated by aldosterone. FE_K_ was related to AKI with fair accuracy and probably a result of a decrease in GFR and aldosterone activation (to maintain potassium homeostasis).

The observed hyponatremia possibly shows volume-overload and is associated with lower urinary dilution, represented by low Uosm. Uosm is the gold standard method for testing urinary concentrations but in medical practice, it is usually estimated by specific gravity in urine or indirect measurements. Sodium is the most abundant ion in the extracellular fluid and the key determinant of extracellular volume. Its plasma concentration is tightly determined, so its management is essential to survival. Hence, the sodium concentration control in urine represents a measure of tubular function. The high urinary sodium concentration in both groups is compatible with renal tubular impairment.

The specific therapy for *Bothrops* envenomation is the antivenom; however, class P-I SVMPs, serine proteases, PLA2 molecules, disintegrins and bradykinin-potentiating peptides show a weak reaction with antibothropic antivenom [21]. The use of fresh frozen plasma in Australian snakebites resulted in more rapid restoration of clotting function in most patients but no decrease in discharge time [43]. The maintenance of serum sodium within the normal range and the control of coagulation disturbances might interfere in AKI development and require further study.

*Bothrops* envenomation caused asymptomatic renal tubular dysfunction, manifesting as the inability to concentrate urine in both the AKI and No-AKI groups. This is the first study describing abnormal urine-concentrating ability in *Bothrops* snakebite. These abnormalities were likely related to renal transporters, such as aquaporin 2 or Na-K-2Cl cotransporters, which have been found to be altered in other tropical diseases [44]. The analysis of urinary transporters might be useful in the detection of this dysfunction at the molecular level, which should be tested in further studies.

## 4. Methods

This is an observational prospective study, which analyzed a subset of patients (n = 63) admitted due to snakebite caused by the *Bothrops* genus. We recruited these patients from December 2015 to December 2016, at the Dr. Jose Frota Institute, a referral emergency hospital in a large urban center of Fortaleza, Ceara, northeast Brazil. The inclusion criteria were patient’s age between 10 and 65 years and confirmed accident caused by *Bothrops* snakebite. Exclusion criteria were confirmed pregnancy, presence of previous kidney disease, diabetes mellitus, hypertension and use of diuretics.

Demographic and clinical data were collected on admission. Patients meeting the inclusion criteria provided their free and informed consent and had ≥2 blood samples collected after being recruited, as described in Figure 1. The novel renal urinary biomarkers MCP-1 and human NGAL in urine and serum were measured on hospital admission. A healthy control group was matched according to age and gender. The study was approved on 5 June 2015 by the Human Research Ethics Committee of the University of Fortaleza (protocol number: 41664214.5.0000.5052).

### 4.1. Sample Collection and Laboratory Assays

Urine and blood samples were collected on hospital admission for measurement of the new biomarkers. Serial samples of serum sCr, Prothrombin Time (PT) and aPTT were collected until discharge. Serum sodium and potassium were determined in an ion-selective electrolyte analyzer (9180, Electrolyte Analyzer, Roche^®^ (Mannheim, Germany) and the results were expressed as mEq/L. GFR was estimated using the CKD-EPI formula for adults and the Schwartz formula for those aged <16 years [45,46].

Patients were classified according to the severity of the snakebite accident using the Brazilian Ministry of Health criteria [7]. AKI was defined based on the Kidney Disease Improving Global Outcome (KDIGO) criteria [20].

Prothrombin Time (PT) and Activated Partial Thromboplastin Time (aPTT) were measured in citrated plasma, 3.8%. All samples were processed on a Sysmex CA-1500 automated blood coagulation analyzer (Sysmex Corporation, Chuo-Ku, Kobe, Japan) using standard coagulometric or immunoturbimetric methods according to the manufacturer’s protocols. PT was defined according to reference values as normal (10–14 s), abnormal (15–129 s) and incoagulable tests (≥130 s). aPTT was defined as normal (22–28 s), abnormal (29–179 s) and incoagulable tests (≥180 s). Total recovery of the blood coagulation status (PT and aPTT tests) up to 24 h after antivenom administration was considered as an efficient dose criterion. Otherwise, an additional antivenom dose (3–6 vials) was administered.

Urinary creatinine was measured in a Cobas C111 analyzer (Roche^®^). Urine total protein excretion was quantified by the colorimetric pyrogallol red method (Labtest^®^, Columbus, Ohio, USA). Urinary total protein was calculated by the urinary creatinine ratio, resulting in a protein/creatinine ratio, expressed as mg/g-Cr. Renal biomarker levels were measured using enzyme-linked immunosorbent assay (ELISA) kits (R&D Systems, Minneapolis, MN, USA). Urinary and serum NGAL (DY1757-Duoset, R&D Systems) and urinary MCP-1 (DY279-Duoset, R&D Systems) levels were all evaluated in duplicate.

The fractional excretion of sodium (FE_Na_) (%) was estimated according to the equation: urinary[Na]_(mEq/L)_ × serum[Na]_(mEq/L)_/urinary[Creatinine]_(mg/dL)_ × serum[Creatinine]_(mg/dL)_ × 100. An analogous formula was used to calculate the Fractional excretion of potassium (K) and the Fractional excretion of Urea (Ur).

Osmolality of urine (Uosm) (*mOsm/kgH_2_O*) was calculated as follows: [(urinary[Na]_(mEq/L)_ + urinary[K]_(mEq/L)_) × 2] + (urinary[Ur]_(mg/dL)_ × 0.16651) + (urinary[Glucose]_(mg/dL)_ × 0.055). Plasma osmolality (Posm) (*mOsm/kgH_2_O*) was calculated using the formula: [(serum[Na]_(mEq/L)_ + 10) × 2] + (serum[Glucose]_(mg/dL)_ × 0.055) + (serum[Ur]_(mg/dL)_ × 0.16651).

Urinary concentration defect was defined as (Uosm)_(*mOsm/kgH2O*)_/(Posm)_(*mOsm/kgH2O*)_ < 2.8 measured on admission in a random sample of urine before any pharmacological treatment. Transtubular Potassium Concentration Gradient (TTKG) was predicted using the formula: urinary[K]_(mEq/L)_/serum[K]_(mEq/L)_ × (Posm)_(*mOsm/kgH2O*)_/(Uosm)_(*mOsm/kgH2O*)_.

### 4.2. Statistical Analysis

Epidemiological, clinical and laboratory characteristics were compared between patients who developed AKI (AKI group) and those who did not develop AKI (No-AKI group). The normality of variables was evaluated using the Shapiro–Wilk Test. Risk factors for AKI were determined by multivariate analysis with logistic regression. Categorical variables were analyzed using the Chi-Square and Fisher’s exact test. Incoagulable and abnormal PT and aPTT were analyzed like categorical variables in comparison with normal tests. Spearman’s correlation coefficients were used to estimate the correlation between biomarkers and kidney functions. One-way ANOVA was applied to compare the AKI group, No-AKI group and the healthy control group. The area under the receiver operator characteristic curve (AUC–ROC) was used to evaluate the diagnostic performance of biomarkers on admission. Analyses were performed using GraphPad Prism v. 7 (Graph Pad Software, San Diego, CA, USA) and SAS/Stat v. 13.2 of the SAS system for Windows.

## Figures and Tables

**Table 1:** Demographic and clinical characteristics of patients admitted after *Bothrops* envenomation according to AKI development.

Characteristic	No-AKI (n = 41)	AKI (n = 22)	*P*
Age (years)	39 (12–86)	42.5 (10–85)	0.39 ^a^
Time between snakebite-medical care (hours)	8 (0.5–72)	6.5 (1–144)	0.85 ^a^
Time between snakebite-antivenom (hours)	10.5 (1–76.25)	9 (4–157)	0.64 ^a^
Hospital stay (days)	2 (0–5)	3 (1–15)	0.003 ^a^
Vials of antivenom (n)	6 (2–12)	4.5 (3–12)	0.39 ^a^
Rural area n (%)	38 (92.7)	21 (95.5)	1.00 ^b^
Male gender n (%)	28 (68.3)	10 (45.5)	0.07 ^c^

^a^ Kruskal–Wallis test. Non-Normality according to the Shapiro–Wilk normality test. Variables are expressed as median, minimum and maximum values. ^b^ Fisher’s Exact Test. ^c^ Chi-Square Test.

**Table 2:** Laboratory parameters of patients admitted after *Bothrops* envenomation according to AKI development.

Variable	No-AKI (n = 41)	AKI (n = 22)	*P*
Mean Hemoglobin (g/dL)	13.4 (8.9–15.4)	12.28 (7.27–14.55)	0.03 ^a^
Lowest Hemoglobin (g/dL)	12.7 (8–15.4)	11.7 (6–13.5)	0.008 ^a^
Mean Hematocrit (%)	38.55 (25.9–44.3)	35.1 (22.07–41.35)	0.019 ^a^
Lowest Hematocrit (%)	37.1 (23.4–44.3)	33.2 (17.4–38.8)	0.005 ^a^
Leukocytes on admission (per mm^3^)	11,025 (5080–21,470)	11,740 (5890–21,870)	0.37 ^a^
Mean Leukocytes (per mm^3^)	10,591 (SD: 2789)	10,595 (SD: 2529)	0.99 ^b^
Lowest Platelets (per mm^3^)	177,075 (SD: 57,756)	162,286 (SD: 93,442)	0.51 ^b^
Platelets on admission (per mm^3^)	194,225 (SD: 63,305)	187,762 (SD: 102,071)	0.79 ^b^
Lowest Serum Sodium (mEq/L)	142 (136–150)	139 (126–147)	0.02 ^a^
Mean Serum Sodium (mEq/L)	143.1 (SD: 3.87)	141.3 (SD: 4.03)	0.09 ^b^
Lowest Serum Potassium (mEq/L)	3.75 (SD: 0.34)	3.73 (SD: 0.26)	0.89 ^b^
Mean Serum Potassium (mEq/L)	3.87 (SD: 0.33)	4.04 (SD: 0.35)	0.07 ^b^
Serum Potassium on admission (mEq/L)	3.88 (3.34–4.7)	4.02 (3.37–5.9)	0.11 ^a^
Mean Creatine Kinase (U/L)	300.5 (47–927)	240.2 (49.17–1854)	0.43 ^a^
Mean Serum Glucose (mg/dL)	99 (54–172)	100 (85–213)	0.59 ^a^
Mean Serum Albumin (mg/dL)	4.1 (SD: 0.4)	4.28 (SD: 0.5)	0.29 ^b^
**Renal parameters**			
eGFR on admission(mL/min per 1.73m^2^)	99.31 (SD: 3.91)	56.45 (SD: 6.99)	<0.0001 ^b^
Baseline Creatinine (mg/dL)	0.8 (0.3–1.5)	0.85 (0.5–2.7)	0.23 ^a^
Mean Serum Urea (mg/dL)	34 (13.5–70)	45.13 (20–153.1)	0.004 ^a^
Serum Creatinine on admission (mg/dL)	0.9 (0.5–1.5)	1.45 (0.7–6.3)	<0.0001 ^a^
Proteinuria (mg/dL)	21.8 (4.7–118.7)	50.9 (6.9–168.9)	0.12 ^a^
Proteinuria (mg/gCr)	260.1 (75–6303)	643 (162–5235)	0.03 ^a^
**Novel Kidney Biomarkers on admission**			
Urinary MCP-1(pg/mgCr)	274.1 (15.1–3562)	547.5 (86.2–5514)	0.02 ^a^
Urinary MCP-1 (pg/mL)	258.2 (24.7–793.1)	447.3 (25.4–1147)	0.01 ^a^
Urinary NGAL (ng/mgCr)	12.7 (0.2–452.5)	21.3 (5.1–99.6)	0.03 ^a^
Urinary NGAL (ng/mL)	10.2 (0.3–21.8)	16.97 (3.09–22.4)	0.004 ^a^
Serum NGAL (ng/mL)	176.5 (SD: 47.1)	181.7 (SD: 58.3)	0.72 ^b^
**Tubular function on admission**			
FE Sodium (%)	0.82 (0.01–6.8)	1.395 (0.22–13.28)	0.08 ^a^
FE Potassium (%)	8.6 (0.02–20.8)	14.5 (5.4–55.5)	<0.0001 ^a^
FE Chloride (%)	1.3 (0.01–11.3)	2.19 (0.59–16.75)	0.04 ^a^
FE Urea (%)	38.79 (0.29–118.48)	48.98 (2.14–66.67)	0.33 ^a^
Uosm, *mOsm/kg*	516.6 (167–972)	383.1 (131.7–852.8)	0.08 ^a^
Posm, *mOsm/kg*	308.7 (271.6–321.4)	310.5 (299.5–377)	0.05 ^a^
TTKG	4.93 (1.39–16.99)	5.75 (2.62–16.74)	0.40 ^a^
Uosm/Posm	1.69 (0.54–3.3)	1.21 (0.35–2.7)	0.08 ^a^
Urinary Sodium (mEq/L)	116.6 (SD: 12.7)	97.18 (SD: 13.5)	0.32 ^b^

Abbreviations: AKI, acute kidney injury. eGFR, estimated glomerular filtration rate using the CKD-EPI formula for adults and Schwartz formula for individuals <16 years old. uMCP-1, Urinary monocyte chemotactic protein-1. uNGAL, Urinary neutrophil gelatinase-associated lipocalin. sNGAL, Serum neutrophil gelatinase-associated lipocalin. FE, Fractional Excretion. Uosm, Urinary Osmolality. Posm, Plasmatic Osmolality. TTKG, Transtubular Potassium Concentration Gradient. Reference Values: Hemoglobin 11.5–18 g/dL; Hematocrit 36–54%; Platelets 150,000–450,000 mm^3^; leukocytes 3600–10,000 mm^3^; Creatinine 0.6–1.3 mg/dL; Urea 13–43 mg/dL; Sodium 135–146 mmol/L; Potassium 3.5–5.3 mEq/L; Magnesium 1.9–2.5 mg/dL; Calcium 8.5–10.5 mg/dL; Creatine Kinase <195 U/L. ^a^ Kruskal–Wallis test. Non-Normality according to the Shapiro–Wilk normality test. Variables are expressed as median, minimum and maximum values. ^b^ Student’s *t* test. Normality according to the Shapiro–Wilk normality test. Variables are expressed as mean and standard deviation values.

**Table 3:** Independent variables associated with AKI development following *Bothrops* envenomation.

Variables	Acute Kidney Injury		
	OR	95% CI	*P*
Lowest Serum Sodium (mEq/L)	0.734	0.57–0.94	0.0160
Lowest Hemoglobin (g/dL)	1.036	0.664–1.616	0.8756
Proteinuria (mg/gCr)	1.0	1.00–1.001	0.3687
aPTT on admission (normal vs. abnormal/incoagulable)	26.272	1.348–512.11	0.031

**Table 4:** Correlation between novel renal biomarkers and renal function parameters.

Novel Renal Biomarkers/Renal Parameters	uMCP-1 (pg/mgCr) *		uNGAL (ng/mgCr) *	
	Spearman’s Correlation Coefficient	*P* Value	Spearman’s Correlation Coefficient	*P* Value
Proteinuria (mg/gCr)	0.70	<0.0001	0.47	0.001
FE sodium (%)	0.44	0.003	0.56	<0.0001
FE potassium (%)	0.15	0.34	0.09	0.56

* uMCP-1 and uNGAL were normalized by urinary creatinine (mg).
